# Hospital Meetings

**Published:** 1903-03-28

**Authors:** 


### HOSPITAL MEETINGS.

### ST. GEORGE'S HOSPITAL.

A special meeting of the Governors of St. George's Hospital was held on March 20th, at Grosvenor House, by permission of the Duke of Westminster, to consider the desirability of removing the hospital to another site. The pros and cons of the suggestion were summed up in two documents, printed on buff and green paper respectively, which were placed in the hands of all the governors.

The chair was taken by Mr. Timothy Holmes, treasurer, in the unavoidable absence, through indisposition, of the Duke of Richmond.

The meeting had been called, said the Chairman, to consider the question of the removal of the hospital. The board had now an offer from the proprietors of four houses in St. George's Place. The purchase of those houses would secure the enlargement of the hospital to that extent only. It was quite impossible to carry on efficiently the work of the hospital and the medical school on the present site. One alternative was to sell their present site, but it should be remembered that they could only sell with the consent of the Charity Commissioners, and most probably an Act of Parliament would have to be passed. Another point was that they were only at liberty to sell part of the site, as a portion belonged to the Westminster estate, from whom they leased it at a nominal rent on condition that it was used for hospital purposes. A tentative offer of £350,000 or £375,000 had been received by the board for the site and buildings, but the names of the proposed purchasers had been withheld, and the board only knew that the offer had been made through a respectable firm of agents. The cost of acquiring a new site of, say, five or six acres would have to be considered, besides the outlay involved in erecting new buildings, which alone might cost £240,000, besides what would have to be paid for the site. If the board purchased the freehold of the four houses they might pull them down and erect higher buildings, and, if the authorities consented, wider, which would give them a considerable amount of additional space. The question must be faced that if the hospital remained where it was large alterations would have to be made, and the purchase of the freehold and pulling down of the buildings would involve them in an expenditure of £170,000, if not more.

Sir William Bennett moved, "That if a suitable site can be found for St. George's Hospital within the area ordinarily served by it, and if the interest of the governors in its present site and buildings can be disposed of to such advantage as to enable them to acquire such new site, and to erect thereon a complete modern hospital establishment of not less than the present number of beds, and to meet all contingencies connected with removal, the governors are ready to consent to the removal of the hospital." The history of the hospital went back, said Sir William Bennett, to the year 1733. In the year 1868 the liberality of the Duke of Westminster enabled the governors to make large alterations and improvements, and it was then taken for granted that in accordance with the circumstances of that time the hospital was then in a complete condition, and sufficient for all purposes. From the year 1891 the need for further accommodation grew greater and greater, and between the dates 1868 and 1902 £190,000 was expended on improving the hospital and increasing the accommodation without at the same time adding one extra inch of space to the available ground on which the hospital stood. The hospital possessed many excellent qualities, and was in many respects as good as, if not better than, any other hospital; but it was entirely wanting in everything that a modern hospital required, and it was quite impossible to carry it on, as it should be, on the lines of modern science. The governors were the holders of a great trust which they should administer to the best of their ability for the benefit of the sick poor, for whom the hospital was originally instituted. No one could maintain that the hospital at present was anything but defective, and however excellent its qualities they were no apology for defects which rendered its proper working impossible. Sir Wm. Bennett then drew the governors' attention to some of the most glaring of the hospital's defects, but went on to say that it was a perfectly healthy building for ordinary purposes, and the results of the treatment of patients compared favourably with those of any hospital in the world. The medical staff of the hospital by a majority of 16 out of 22 were in favour of removal. It was calculated that quite 45 per cent. of the patients would be actually benefited by the removal of the hospital to Fulham, where there was a population of 150,000 without any hospital accommodation whatever. To gain room by sacrificing 30 or 40 beds, as was suggested, would be a most disastrous step to take. In conclusion, Sir William Bennett said the resolution was only tentative, merely suggesting the removal of the hospital to another site if the necessary money were available.

The resolution was seconded by the Hon. Dudley Fortescue.

Dr. W. H. Dickinson proposed as an amendment, "That it would be desirable to proceed with the negotiations for the purchase of the four houses in St. George's Place, and to make such arrangements as may be practicable with regard to that part of the present site which is held of the Westminster estate." Dr. Dickinson was of opinion that one of the results of moving the hospital, say to Fulham, would be to alter the class of medical students attending the hospital, another drawback being that the poor would not be so much brought into contact with the governors as heretofore.

Sir William Karslake, K.C., seconded the amendment on the grounds that he did not wish to see an interregnum during which St. George's Hospital would not exist. The purchase money of the freehold could not be touched till the hospital was vacated. The cost of removal and erecting new buildings could not be put down at less than £300,000, and possibly it might amount to £500,000.

The Chairman, in supporting the amendment, said Sir William Bennett's resolution entirely depended on an if—*if* they could get the necessary money. Mr. Holmes was of opinion that the Charity Commissioners would not grant leave to sell the freehold unless the governors were unanimous.

Sir Isambard Owen, who spoke in favour of the resolution, said that unless they could get a fancy price for the freehold and buildings they had no intention of moving, but he thought that the possession of the four houses in St. George's Place was absolutely essential in any case.

Sir Henry Burdett said he fully recognised the difficulties presented by the problems under discussion. He considered the meeting was not in a position to vote wisely and definitely for either the resolution or the amendment for lack of accurate data based upon inquiry and backed up by expert knowledge. Sir Henry then proposed another amendment, which he thought would bring them all into line, to the effect that twelve governors should be chosen as a special committee to inquire into the whole matter and to report in two months' time. Out of 351 beds in the hospital there were 133 which Sir Henry said as an expert he must describe as bad beds.

Effect was subsequently given to this amendment, and an alteration was made, according to Sir Henry Burdett's suggestion, in the wording of Sir William Bennett's resolution, to the effect that the governors were ready to "consider" instead of "consent to" the removal of the hospital, and also in the subsequent resolution appointing a committee.

The discussion was continued for some time, and then Dr. Dickinson's amendment was voted upon by a show of hands and was rejected by a majority of 51 to 32. Sir William Bennett's resolution as amended was carried by a majority of 58 to 8. A committee was then appointed to inquire into all the facts and questions involved, to treat with intending purchasers, and to negotiate for the purchase of a new site.

Amongst those present at the meeting were Lord Rowton, Lord Frederick Fitzroy, Lord Elcho, Lord Barnard, the Dowager Countess of Shrewsbury, etc., whilst letters of apology were read from the Earl of Wemyss, the Earl of Clanwilliam, the Marchioness of Londonderry, and Viscount Portman.

### SAMARITAN FREE HOSPITAL.

The annual meeting of Governors of this hospital was probably the briefest on record: it was held on Tuesday, the 10th instant, and lasted barely seven minutes. The Chairman was Dr. Oxford, and six other Governors attended. The report and accounts were taken as read and adopted without comment; the officers for the ensuing year were elected in the same rapid manner; and a vote of thanks to the medical staff and another to the chair concluded the sitting. The falling in of the leases of adjoining property during 1903 and 1904 will enable the committee to take active steps to remodel the out-patients' department in accordance with its needs and with the recommendation of the visitors for King Edward's Hospital Fund. A sum of £5,000 will be needed to defray the cost of this reorganisation, and donations to the new building account, which has already been opened, are requested for this purpose.

### ROYAL LONDON OPHTHALMIC.

New Premises Paid For and The Building Account Closed.

The annual general meeting of Governors of the Royal London Ophthalmic Hospital, was held on the 4th instant, Mr. H. P. Sturgess, the chairman, presiding.

He read letters of regret for non-attendance from Lord Avebury and Lord Hillingdon, the Bishops of London and Stepney, and other gentlemen, and then proceeded to deal with the report and accounts for the past year. It was a great pleasure to the Committee of Management, he said, to be able to report that the hospital was in a thoroughly efficient condition. He could not lay too much stress on the absolutely harmonious way in which all connected with the institution worked together. That the hospital was needed was proved by the increased number of patients treated. Through the special grant made by King Edward's Fund for the purpose, they had been able to open 18 more beds and these had been occupied throughout the year. Since then they had opened a further 30 beds, the King's Fund again making a special donation of £2,500 for the purpose, and increasing the annual grant to £2,000. These beds were opened by the Lord Mayor, and he desired to express their thanks to his lordship for the wise words he uttered on that occasion. Those beds also were immediately filled and so, on the previous night, they had had 101 patients sleeping in the hospital. Well, it might be asked, if they had an efficient and well-managed hospital, and patients who wanted to make use of it, what more did they want? They wanted money to carry on the work. They had a deficit of £5,169 at the beginning of the year, and that had been increased to £5,463. In 1901 they were only able to keep open by borrowing £5,000 from the Charity Commissioners, and as against that loan the Commissioners held securities valued at £7,000, retaining the interest on them towards repayment. Everything that was possible was done to reduce expenditure, and the committee were constantly examining every item and detail to see what they could save; but without interfering with efficient working, he could hold out no hope of further curtailment. Their rates were nearly £1,000 a year, and steadily increasing. They felt very strongly that hospitals generally should be relieved of rates, and were taking steps with other hospital authorities with a view to bringing this about. Their ground rent was also very heavy—£1,210 a year—and for that they were starting an investment fund. As to their income, donations showed a slight increase, but subscriptions, he was glad to say, showed a substantial advance. Comparing those of 1898 with last year they would see they had very nearly doubled during that time—a most hopeful sign. Just at the close of the year they received a legacy of £2,000, and that they proposed to invest. It was good policy to treat legacies as capital and invest them, and they hoped they would not have to realise the money for current expenses. Another important item was the contributions from patients. Every patient had the opportunity of subscribing, but not the slightest pressure was put upon them, and every subscription was absolutely free and voluntary. Summing up the position it was this: They had a total expenditure of close on £12,000, which would be increased in the current year on account of the extra beds that had been opened. Their assured income from annual subscriptions and King Edward's Fund was about £3,700. If the grants from the three hospital funds were the same as last year, £3,500, that would be £7,000, so that they would require a further £6,000 to make ends meet. They were also anxious to get donations to the special fund to repay the loan of £4,700 and so release their securities, and that of £50,000 to guarantee their rent, to which about £1,200 had already been subscribed. The fact that the King's Fund was paying them altogether some £4,500 a year was proof of their efficiency, for it was well known that that was a special point with them, and they therefore appealed confidently for help. He must not omit to mention the great assistance that had been received from the Royal London Ophthalmic Hospital Guild which provided them with linen as well as obtaining subscriptions, and to which they therefore owed most cordial thanks. He moved the adoption of the report and accounts.

Mr. Davison, the deputy chairman, seconded, and went at some length over the items in the accounts, drawing attention to the final balance-sheet of the building fund account, which was now closed by the transference of the balance of nearly £600 to the rent fund. The building committee had now been dissolved as no longer necessary. He pointed out that unlike many other hospitals they presented not only an income and expenditure account, but a balance-sheet as well —the only true test of a hospital's position. He emphasised the need for relief from rates, theirs being, he said, greater than those of any other hospital except St. Thomas's and Guy's. Next year would be the centenary of the Royal London Ophthalmic, and he appealed to wealthy donors to mark that special occasion by helping them with one or other of their special funds.

Sir John Bell, in proposing the re-election of the retiring members of the committee of management said he hoped in striving to keep down expenses they would not sacrifice efficiency to economy.

Other formal business concluded the meeting.

---

### NATIONAL HOSPITAL FOR THE PARALYSED AND EPILEPTIC.

The annual general meeting of this hospital was held on March 12th, the Rev. Prebendary Wace, D.D., in the chair.

The chairman said the past year had been necessarily one of some anxiety to those who were comparatively new to the character of the hospital. He thought that the general condition of the hospital was satisfactory and that its financial position was improved. The efficiency of the hospital had been much increased in consequence of their having been able to carry out the recommendations of the committee of inquiry. The heating, lighting, and drainage of the hospital had been very materially improved. They had been fortunate in their finances during the last year owing to a large number of legacies having been received. In consequence of the untoward circumstances of last summer they had been unable to hold their festival dinner, and therefore they estimated that they had lost from £2,000 to £3,000. Dr. Wace went on to congratulate the hospital on the successful working of the new constitution—they had experienced no difficulties in carrying it out, but had found the co-operation of the medical staff most satisfactory to the best interests of the hospital. Neither had they found any difficulties arise from the threefold administration of the secretary, senior house physician, and the lady superintendent, who all worked under the board. The chairman said that the committee felt how much the work of the hospital had been facilitated by the kindness of Mr. Danvers Power, their vice-chairman, in discharging in an honorary capacity the duties of secretary for the first two months of the new system.

Dr. Buzzard said he spoke in the name of the medical staff to assure the committee how grateful they felt for the promptitude with which the board of management had taken into consideration all such alterations as were necessary to bring the hospital into a state of efficiency. The introduction of electric light was an enormous improvement to the hospital; it was particularly requisite, having regard to the type of disease in their hospital, to get the electric current brought to the bed of each patient. An outside fire escape had also been installed, a necessity which ought long ago to have been provided, but which the present board had lost no time in instituting. In conclusion Dr. Buzzard stated that nothing could exceed the harmony which existed between the board and its medical officers.

Mr. Burford Rawlings, who was the next speaker, offered a few comments on the empty state of the room, and said it completely refuted the charge made against the late board of keeping away the Governors from the meeting. The late board had acted in the same way as the present, and had sent out notices, etc., but with very little success. Proceeding, Mr. Burford Rawlings said with regard to the outside fire appliances that the late board had taken the best advice at the time the hospital was built and had never afterwards had any suggestion made to them that anything of the kind should be done. As regards the other improvements, every one of them had been under the consideration of the late board, and would have been carried out as soon as the lease of the two adjoining houses fell in and thus gave them the required opportunity. Mr. Burford Rawlings took exception to the statement in the report that the financial position of the hospital was improved, on the grounds that, although the receipts for the last year were larger than they had ever before been, yet the greater part of this sum was made up of amounts which were standing over owing to legal procedures, and that there was also one large legacy which had been bequeathed to the hospital during the tenure of the late board. He also criticised adversely the realisation by the board of some of the capital funds in order to defray the cost of the alterations. The expenses also had risen considerably, the cost per bed being now £96.

The Vice-Chairman, Mr. J. Danvers Power, in reply to Mr. Burford Rawlings, said that the statement in the report to the effect that the financial position of the hospital was improved did not at all mean that they claimed to have been more successful owing to their own efforts or merits than the late board; in fact they had pointed out that they were extremely fortunate owing to the appeals made during the *régime* of the late board. It was extremely difficult to say what the financial position of the hospital was, and the only real method of gauging that position was to set down the assets and liabilities. With regard to their having withdrawn a sum from capital, they had taken the best advice on this point, and if they had not withdrawn that sum, as it was their legal right to do, they would have been obliged to take the money out of other funds of the hospital. As to economy, immediately on taking office the present board had asked for tenders for everything supplied to the hospital, which was a new departure in its management. If the board had attempted any economy in the amount of food supplied to the patients, they would have had to take the responsibility of refusing to follow the advice of their medical committee. It was very easy to say that the sum of £96 per bed was very high, but it was not so easy to point out how economy could be effected.

The Chairman remarked that the apparent increase in the cost per bed was partly accounted for by the fact that, owing to the alterations, a certain number of beds were unoccupied.

The meeting then became a special general meeting in order to consider the question of a petition to the King for the grant of a Royal Charter and other points.

Sir Felix Semon set forth the great advantages the hospital would gain by being incorporated under a Roya Charter, and stated that as they were now situated they had no legal status. This difficulty had been brought before them during the last year in connection with their Home at Finchley, when negotiations for acquiring land had fallen through on this account.

Mr. W. W. Paine, the honorary solicitor of the hospital, and Sir John Paget having further explained the various clauses of the petition, a unanimous vote was passed approving the draft petition proposed to be presented to the King for a Royal Charter of incorporation.

---

### ROYAL HOSPITAL FOR DISEASES OF THE CHEST, CITY ROAD.

Notwithstanding repeated insistence, both by the Council of the King's Hospital Fund and by the medical staff of this hospital, on the necessity for the nurses' home, the consideration of the plans has had to be postponed for lack of funds to carry them out. It was on this point that the Lord Mayor laid stress in his opening remarks on the occasion of the annual Court on March 19th. The hospital, he said, had submitted itself, voluntarily, of course, to the investigations of the very able and experienced gentlemen who were responsible for the administration of the King's Fund, and the mere fact that £500 had been received from them for the new building was a sufficient guarantee of the necessity for it. The managers, moreover, agreed with the Council and the medical staff, and there was no question that the claims of those who tended the sick should have full consideration. Women who gave their time and strength to aid the doctors in the alleviation of sickness should have suitable opportunities for rest and refreshment, especially in a hospital where the nature of the diseases treated rendered pure air more than ever essential to the maintenance of health among the staff. It was for this object that the managers were obliged to appeal in the letter signed by Mr. S. Hope Morley, treasurer, and Sir T. Andros de la Rue, chairman, a copy of which was in the hands of all who attended the meeting. There was another great need, and that was a country branch, and for this object also the managers appealed. The medical staff were unanimously agreed that both were absolutely necessary, and the managers were only waiting for funds to enable them to proceed. A freehold site for the nurses' home had already been purchased at a cost of £2,000. For his own part, and that of the Lady Mayoress, he was prepared to give £21 towards the special fund, and he sincerely hoped that the managers of this fine old institution would very soon be out of their financial difficulties.

The hospital had received a certificate of good conduct, without which no charitable institution to-day could or ought to successfully appeal to the public for subscriptions, and he strongly urged its claims. Many of the patients came from outside London, and daily, as he became more experienced in hospital work, he found that this fact was insufficiently recognised: some 25 per cent. were country patients, who came from localities which scarcely contributed a shilling to their support.

### The Need for a Country Branch.

Although Mr. George Berry was the only speaker who alluded in so many words to a statement that "the hospital was in the wrong place," denying emphatically that this could ever be, nearly all the speakers showed that the need of a country branch, if the work is to be satisfactory, is becoming urgently felt. If the open-air treatment was proved to be best, said Dr. Gilbart Smith, this hospital would be second to none in securing it, and he had every confidence that the managers would prove themselves capable of dealing with the question. At the same time he insisted that the cases treated were not incurable or even necessarily consumptive; the hospital existed for the treatment of diseases of the chest, and patients were frequently sent away from the City Road Hospital with a new lease of life. Mr. John S. Gilliatt, in seconding the adoption of the report, said in view of increased knowledge it was scarcely necessary to remind his hearers of the need for open-air treatment. It was possible to do something in a hospital built some years ago, and situated in the metropolis; but, if the treatment were to be carried out with satisfaction to the medical staff, it was essential that there should be a country sanatorium to which patients should go to complete the cure. It was of the greatest importance that the £50,000 needed for this and for the nurses' home should be provided as quickly as possible; without a country sanatorium the work could not be as complete as it ought to be. The Hon. Claude Hay said all institutions of this kind were going through a great crisis, and those who managed them in these days had responsibilities thrown upon them which were no light task; a slight mistake might bring a round of trouble. As one who knew the locality, he could say that it was not possible to measure the good done by this hospital to that particular part of London; he would be wrong and ungrateful were he not to take this opportunity of tendering the thanks of his constituents to the hospital.

### ROYAL NATIONAL HOSPITAL FOR CONSUMPTION, VENTNOR.

In the absence of the Earl of Rosebery, who had hoped to preside at the annual meeting of this hospital, on March 19th, at the Whitehall Rooms, Hôtel Métropole, the chair was taken by Mr. P. B. Burgoyne, chairman of the Board of Management.

The chairman said he was happy to inform the governors that their Majesties the King and Queen had graciously consented to become patrons of the hospital in succession to the late Queen Victoria, and that a Royal visit of inspection had been made during the year. H.R.H. Princess Henry of Battenberg, Governor of the Isle of Wight, also honoured the hospital by a visit in April 1902. Alluding to statistics given at the recent meeting of the National Association for the Prevention of Consumption, the chairman said that 15½ per cent. of the work being done for consumptives in the United Kingdom was being done at Ventnor, where there were 154 beds, 94 for men and 60 for women. The patients spent 22 out of the 24 hours in the open air. During 1902 each patient remained on an average 69 days, equal to 10 weeks. In view of the long waiting necessary in very many cases it was proposed that the qualifications of governors should be raised, so as to restrict the number of letters issued, and he would urge upon the governors the absolute necessity of not giving letters except in cases where benefit was likely to result. With regard to finance, the hospital was £2,000 to the good, having received £16,432 and spent £14,454; £2,500 had been invested. A special want just now was outdoor games for the patients, such as bowls, croquet, etc. He had received a letter from Dr. W. M. Davidson, the resident medical officer, calling attention to the daily increasing difficulty in obtaining employment for patients after leaving the hospital; even their friends refused to mix with them. He appealed to the governors to look after the patients after treatment, and to see that, as far as possible, the conditions necessary for recovery were continued.

A resolution affecting the bye-laws which relate to the privileges of governors was proposed by the deputy chairman, Mr. G. T. Powell. No vested interests, he explained, would be interfered with, since the alterations proposed would apply only to new subscribers and donors. The qualification for becoming a governor would be fifty guineas instead of thirty, while for a donation of £500 (instead of £350) the privilege of having a patient always in the hospital would be granted. Other alterations were proportionate. It was desired to treat patients whose disease was of a curative character, and to get them in as early as possible.

The resolution having been adopted, Dr. T. A. Ross, one of the Ventnor physicians, gave an interesting address on the statistics of the hospital; it was most difficult, he said, to arrange the results of treatment. He deprecated the use of the word "cure": it was not used at Ventnor, since to label a case as cured would necessitate years of observation. Length of stay was in many cases a most important point for the governors to consider in the future.

A former patient, Mr. Dickens, now secretary of the Parcel Postmen's Hospital Fund, and a governor of the Hospital, urged the need of extending the accommodation so as to prevent the long waiting of urgent cases.

The chairman promised that the matter should have consideration, and the meeting adjourned.

### FRENCH HOSPITAL AND DISPENSARY.

The annual meeting of the French Hospital was held on Thursday March 12th. The chairman, Dr. Vintras, after commenting on the satisfactory condition of the hospital generally, in his concluding remarks said that he had seen a recent speech of Sir Henry Burdett's in which allusion was made to the unsatisfactory condition of certain hospitals. He hoped that Sir Henry Burdett would favour them with a visit at the French Hospital in order to be able to assure them that there was nothing unsatisfactory about the condition of their hospital.

From the report for 1902 it appeared that the hospital had admitted 875 in-patients, and had treated 5,009 out-patients. The receipts for the last year showed a decrease of £248, while there was an increase in expenditure of £927, which was due to necessary repairs. Although the hospital receives a greater proportion of French people, yet it is cosmopolitan in character and renders assistance to all strangers who speak French. The cost of each bed is £66, and of each out-patient 1s. 11¾d.

Attention is drawn in the report to the annual dinner to be held at the Hôtel Cecil on April 25th, when His Excellency the French Ambassador will be in the chair. The Lord Mayor and Sheriffs have also promised to attend.

### A BRITISH SANATORIUM IN DAVOS.

A well-attended meeting of British visitors and residents at Davos was recently held in the Assembly Hall of the Hotel Belvedere for the purpose of expressing local opinion on the proposal to erect a British sanatorium for English-speaking consumptives of limited means.

The chair was occupied by Dr. W. R. Huggard, British Vice-Consul at Davos, who in the course of his remarks said that "while recognising the good work done in the past by the Davos Invalids' Home it is felt that the present building is inadequate, and that it is most desirable that it should be replaced as soon as possible by a more suitable institution for English-speaking peoples."

A local committee was formed to assist the London Committee in the establishment of such a British sanatorium. During the meeting the sum of £350 was subscribed. Two donations of £500 having been previously given by visitors to Davos. The Queen, who is patroness of the home, has sent £25.

Dr. William Ewart, 33 Curzon Street, W., is hon. secretary to the London Committee.

### METROPOLITAN CONVALESCENT INSTITUTION.

The annual meeting of the Governors of the Metropolitan Convalescent Institution was held at the office, 32 Sackville Street, under the presidency of Lord Portman, among those present being Mr. M. O. FitzGerald, Mr. Holmes, Mr. A. R. Macdonald, Mr. S. Osborn, and Mr. Alfred Willett. The annual report, which was read by Mr. A. Hayes, the secretary, stated that during the past year no fewer than 6,293 patients were admitted to the homes entirely free of charge from the hospitals or after illness at home—this being the largest number ever received. The board reported that they had completed the purchase of a plot of four and three-quarter acres at Little Common, Bexhill, as the site of the new home for men to be erected with funds left to the institution by the late Mr. Henry Vaughan, and that Mr. Rowland Plumbe, the architect of the London Hospital had been appointed architect for the building, which, it was hoped, would be commenced in the spring. Meanwhile, to relieve the pressure upon the existing Bexhill home, a house at St. Leonards had been taken for a short term, and was opened in June last, with accommodation for 40 women.

The statement of accounts showed a total expenditure of £15,361, of which £12,511 was for the maintenance of the three homes situated respectively at Walton-on-Thames, Broadstairs, and Bexhill, and the temporary home at St. Leonards, and the balance of £2,849 represented the cost of repairs, etc., at Walton, and furnishing the house at St. Leonards. The income was £12,681, leaving a deficit of £2,680. The report concluded with an earnest appeal for additional support.

